# The gp120 Protein Is a Second Determinant of Decreased Neurovirulence of Indian HIV-1C Isolates Compared to Southern African HIV-1C Isolates

**DOI:** 10.1371/journal.pone.0107074

**Published:** 2014-09-04

**Authors:** Vasudev R. Rao, Ujjwal Neogi, Eliseo Eugenin, Vinayaka R. Prasad

**Affiliations:** 1 Department of Microbiology and Immunology, Albert Einstein College of Medicine, Bronx, New York, United States of America; 2 Division of Clinical Microbiology, Department of Laboratory Medicine, Karolinska Institutet, Stockholm, Sweden; 3 Public Health Research Institute (PHRI), New Jersey Medical School, Rutgers The State University of New Jersey, Newark, New Jersey, United States of America; 4 New Jersey Medical School, Rutgers The State University of New Jersey, Newark, New Jersey, United States of America; University of South Carolina School of Medicine, United States of America

## Abstract

Regional differences in neurovirulence have been documented among subtype/clade-C HIV-1 isolates in India and Southern Africa. We previously demonstrated that a C31S substitution in Clade-C Tat dicysteine motif reduces monocyte recruitment, cytokine induction and direct neurotoxicity. Therefore, this polymorphism is considered to be a causative factor for these differences in neurovirulence. We previously reported on the genotypic differences in Tat protein between clade-C and rest of the clades showing that approximately 90% of clade-C HIV-1 Tat sequences worldwide contained this C31S polymorphism, while 99% of non-clade C isolates lacked this Tat polymorphism at C31 residue (Ranga et al. (2004) J Virol 78∶2586–2590). Subsequently, we documented intra-clade-C differences in the frequency of Tat dicysteine variants between India and Southern Africa, as the basis for differential disease severity and showed the importance of the Tat dicysteine motif for neuropathogenesis using small animal models. We have now examined if determinants of neurovirulence besides Tat are different between the clade-C HIV-1 isolates from Southern Africa and India. Envelope glycoprotein gp120 is a well-documented contributor to neurotoxicity. We found that gp120 sequences of HIV-1 isolates from these two regions are genetically distinct. In order to delineate the contribution of gp120 to neurovirulence, we compared direct *in vitro* neurotoxicity of HIV-infected supernatants of a representative neurovirulent US clade-B isolate with two isolates each from Southern Africa and India using primary human neurons and SH-SY5Y neuroblastoma cells. Immunodepletion of gp120 of both US clade B and the Southern African clade C isolates revealed robust decreases in neurotoxicity, while that of the Indian isolates showed minimal effect on neurotoxicity. The gp120 as a cause of differential neurotoxicity was further confirmed using purified recombinant gp120 from HIV isolates from these regions. We conclude that *gp120 is one of the key factors responsible for the decreased neurovirulence of Indian clade C HIV-1 isolates when compared to South African clade C HIV-1*.

## Introduction

HIV associated neurocognitive disorders (HAND) represent a spectrum of neurological disorders ranging from the mild asymptomatic neurocognitive impairment (ANI), the intermediate mild neurocognitive disorder (MND) to the severe HIV associated dementia (HAD). More than half of all HIV-1 infected individuals suffer from neurological consequences due to HIV-1 infection [1]. Although the milder forms of HAND have been determined to be present at significant levels worldwide, the prevalence of HAD has been documented to be significantly low (0%–6%) in India [2], [3] compared to US (15–30%) [4], [5]. Understanding the viral genetic differences responsible for such regional differences in the prevalence of HAD is key to devising better therapeutic strategies to treat HAND by targeting the appropriate viral proteins and pathways responsible.

There have been a series of efforts to study the specific viral determinants that may be responsible for regional differences in disease prevalence. HIV-1 Tat and gp120 are the two most dominant viral proteins that contribute to HIV neuropathogenesis. We previously reported that the Tat protein in 88% of HIV-1C isolates worldwide have a C31S polymorphism, which disrupts a dicysteine motif that is highly conserved (99%) in all the other clades [6]. This polymorphism causes a significant reduction in monocyte chemotaxis induced by both HIV-1C infected macrophage medium and recombinant HIV-1TatC protein [6], [7]. We, along with several other groups, have shown that the C31S polymorphism in HIV-1 Tat-C is also responsible for a compromised ability of Tat to induce proinflammatory chemokines/cytokines [8]–[12] and to cause direct neurotoxicity [11], [13]. Further, using an in-vivo SCID-HIV encephalitis model, we showed that HIV-1C isolate from India causes lower cognitive dysfunction compared to US HIV-1B isolate [12]. In contrast to the low incidence of HAD reported in India and the in vitro studies summarized above that suggested lower neurotoxicity phenotype for HIV-1C isolates, recent cohort studies in Southern African countries where HIV-1C is predominant, reported a higher incidence of HAD. A study in Botswana reported that 38% of 120 HIV+ individuals had some type of neurocognitive impairment, even though most of them were on ART. In South Africa, Joska *et. al* found that 25% of HIV+ individuals had HAD [14]–[17]. *These studies revealed a disparity between findings in India and Southern Africa.* In order to resolve this apparent inconsistency, we evaluated the percentage of HIV isolates containing Tat-CC motif in India, Bangladesh, Zambia, Botswana and South Africa. We found that the low incidence of HAD in India correlates with the low percentage (1–2%) of isolates with Tat-CC motif in India and Bangladesh, while the higher incidence of HAND in Southern African countries correlates with a higher percentage (20–26%) of Tat-CC isolates in South Africa, Botswana and Zambia [18]. These studies indicated that although clade C predominates in both India and Southern African countries, there are *intra-clade Tat genetic differences that can lead to different clinical outcomes* and emphasizes the need for a closer look at polymorphisms in key viral neurotoxic genes when comparing neurocognitive differences. Indeed, our efforts to measure the contribution of HIV-1C to cognitive dysfunction using SCID-HIV encephalitis mouse model showed that the Indian HIV-1C caused minimal effects, while a Southern African HIV-1C resulted in robust neurocognitive defects. Furthermore, we showed that the Tat protein from the Southern African HIV-1 isolate displayed robust in vitro neurotoxicity, while that of Indian HIV-1C was minimal [18] suggesting that differences in Tat dicysteine motif played a key role in the intra-clade, differential neurovirulence.

Besides Tat, another important viral determinant implicated in the pathogenesis of HAND is gp120. HIV-1 gp120 is the viral attachment protein expressed on the surface of virions and infected cells, and it is non-covalently bound to a transmembrane viral protein gp41. Neurons in the brain are exposed to both the gp120 trimers present on virion/cell membranes as well as to soluble monomers or trimers of gp120 shed into the extracellular medium. HIV-1 gp120 binds to NMDA receptors [19] and CXCR4/CCR5 [20] receptors on neurons and causes neuronal apoptosis. It drives the production of excess glutamate [21] from neurons, and also interferes with the ability of dopaminergic neurons to reuptake dopamine [22], thus interfering with two most critical neurotransmitter mechanisms in the CNS. There have been efforts to identify HAD signatures of gp120 protein. In order to identify amino acid signatures in the HIV-1 *env* gene predictive of HAD, Holman and Gabuzda developed a machine-learning approach and examined *env* sequences from 40 HAD and 38 non-HAD patients. They identified 5 signatures associated with HAD diagnosis, which were found in the majority of brain HIV sequences (8 out of 10 HAD patients) examined in an independent cohort [23]. However, there are currently no reports on determinants that are associated for intra-clade, regional differences in HAD incidence.

In this report, owing to the importance of gp120 in HIV neuropathogenesis, we have investigated whether differences in gp120 of HIV-1C isolates from Southern African countries and India account for the differential neurovirulence of viruses circulating in these countries. Phylogenetic analysis of gp120 sequences of HIV-1C isolates from Southern African countries (with high HAD prevalence) and those from India (which has low HAD prevalence) showed that they cluster separately. We further examined the *in vitro* neurotoxicity profiles of HIV-infected supernatants from multiple isolates from these two geographically distinct regions and found that the neurotoxicities paralleled clinical outcomes mentioned above – Southern African isolates showed high neurotoxicity similar to US clade B, while the Indian isolates showed minimal toxicity. The contribution of gp120 to the neurotoxicity of these supernatants was assessed by immuno-depletion of gp120 from infected cell supernatants, and further corroborated by using recombinant purified gp120 proteins of HIV isolates from the two regions, which showed the same differences. These results show that the reduced incidence of HAD in India and the reduced neurocognitive deficits documented in SCID-HIVE mice using the Indian HIV-1C isolates previously are not only due to polymorphism in Tat, but also gp120 protein. This is the first documentation of intra-clade C, regional differences in the neurotoxicity caused by HIV-1 envelope glycoprotein gp120.

## Materials and Methods

### Phylogenetic analysis

gp120 sequences were obtained from the Los Alamos HIV-1 database. The amino acid sequences were generated by translation and were aligned using Clustal-W. All available HIV-1C sequences were retrieved from Los Alamos HIV-1 database (accessed on 13 September 2013). After excluding, from each individual, duplicate sequences and sequences with premature stop codons in the open reading frame (ORF) or frame-shifts, a total 878 HIV-1C sequences from Southern Africa [South Africa (n = 683), Zambia (n = 110)] and South Asia [India (n = 64), China (n = 20) and Myanmar (n = 1)] were obtained for phylogenetic analysis. HIV-1B isolates, ADA, R3B, BAL and SF-162 were used as out-group in the analysis. Neighbour-Joining phylogenetic tree was constructed in MEGA v6.06 with 100 bootstrap supports [24]. The evolutionary distances were computed using the p-distance method. The evolutionary distances were computed using the Maximum Composite Likelihood method. A separate phylogenetic analysis identified the Chinese and Myanmar HIV-1C strains selected were of Indian origin, thus used in the analysis. In addition, the isolates used in the functional in-vitro studies were also included in the analysis.

### HIV-1 isolates and Infection

HIV-1_ADA_ (US clade B), HIV-1_IndieC1_ (Indian clade C isolate) and HIV-1_1084i_ (Zambian clade C) viruses for use in the in vitro neurotoxicity studies were generated by transfection of molecular clone DNA into 293T cells and the virus released was quantitated by p24 Enzyme-linked immune-sorbent assay (ELISA; Applied Biosystems, Inc). The Indian clade C isolates HIV-1_93IN101_ (from which the HIV-1_IndieC1_ molecular clone was generated) and HIV-1_93IN905_ as well as the Zambian HIV-1C isolate, HIV-1_97ZA012_, were all obtained from NIH AIDS Reagent Program, Division of AIDS, NIAID, National Institutes of Health. Elutriated primary human monocytes and peripheral blood mononuclear cells (PBMCs) were obtained from the University of Nebraska Medical Center. These were collected under the UNMC IRB approved research protocol (No.162-93-FB) with the title: ‘*Leukapheresis of Normal Donors for Use in Studies of Disease Pathogenesis and Therapy*’. Informed written consent was obtained in both of the above studies. PBMCs were activated with PHA and IL-2 prior to infection with HIV-1. For neurotoxicity tests using HIV-1-infected monocyte-derived macrophage (MDM) supernatants, Monocytes were differentiated into MDMs and infected with HIV-1_ADA_ (US clade B), HIV-1_IndieC1_ (Indian clade C) and HIV-1_1084i_ (Zambian clade C). For all *in vitro* experiments, a comparable level of virus production was achieved both in MDM and PBMC cultures among different viruses in each set of viruses tested by varying the multiplicity of infection. Equivalence of virus levels between cultures was determined both by measuring the p24 levels in the infected cell supernatants (for HIV-infected MDMs, we found that the p24 levels ranged from 64 ng/ml to 79 ng/ml for HIV-1_ADA_, HIV-1_IndieC1_ and HIV-1_1084i_ isolates) and by p24 immuno-staining of HIV-infected MDMs (which showed that approximately 33% to 36% of MDMs were p24 positive) [7], [12].

### Immuno-depletion of gp120

The gp120 was immune-depleted from HIV-1 infected MDM and PBMC supernatants using broadly neutralizing anti-gp120 antibodies (VRC 03) (Wu et al., 2010) as described earlier. For this purpose, anti-gp120 antibodies were first bound to Pansorbin (Calbiochem, Cat no. 507861) by incubation on ice for an hour. Subsequently, the antibody-pansorbin complexes were centrifuged and resuspended in 25 µl DMEM and added to spent medium from the HIV-infected MDM. Following incubation for an hour, gp120-antibody-Pansorbin complexes were centrifuged to remove gp120. This step was repeated one more time to ensure complete removal of gp120 from the infected medium. The medium after pelleting the Pansorbin complexes for the second time was then used for the *in vitro* neurotoxicity assays.

### Recombinant gp120 proteins

Recombinant, purified gp120 proteins (greater than 90% pure according to manufacturers) from HIV-1_Bal_, HIV-1_R3B_ and HIV-1_96ZM651_ were obtained through the NIH AIDS Reagent Program, Division of AIDS, NIAID, NIH: gp120 from DAIDS, NIAID. HIV-1_IN16055_ recombinant gp120 was purchased from ACRO Biosystems, Elk Grove, CA.

### Neurotoxicity assays

#### Primary human neurons

Human neurons were obtained from aborted human fetal cortical tissue for the Einstein human tissue repository as part of an ongoing research protocol approved by the Albert Einstein College of Medicine and Rutgers University (Protocol numbers: 02-21-12-01A1 and 2012001303). Written informed consent was obtained from the mothers for the human fetal tissue repository in concordance with the institutional review board of the Albert Einstein College of Medicine. Primary Human Neurons were isolated and cultured as described previously in Neurobasal media plus N_2_ supplement, 1% fetal bovine serum and 1% Pen/Strep. Media was replaced every 3^rd^ day [18]. From this culture, using minimal trypsinization technique, neurons were selectively removed and approximately 100,000 primary neurons were plated on coated MatTek well plates for a period of 2–3 days and allowed to stabilize and differentiate. For experiments involving HIV-1 infected MDM cultures, 100 µl of infected supernatant/infected gp120-neutralized supernatants were diluted with 100 µl of Neurobasal medium and then added to primary Neurons. For testing the neurotoxicity of recombinant gp120, gp120 proteins diluted in PBS were added to 200 µl of Neurobasal medium to achieve a final concentration of 1 nM. Following incubation for 18 hours at 37°C with the infectious supernatants or for 24 hours at 37°C with the recombinant gp120 s, neurons were fixed and TUNEL assay was performed using the TMR in situ hybridization kit (Roche, Switzerland). Neurons were also stained using anti-neurotubulin antibodies (Abcam, USA) and 4′,6-diamidino-2-phenylindole (DAPI; Prolong gold anti-fade agent with DAPI (Invitrogen, USA) to distinguish them from astrocytes. Image capture and analysis was done using Nikon (NIS elements) advanced research software. The results were plotted from triplicate wells from two different sets of neuronal cultures.

#### SH-SY5Y Cells

SHSY-5Y neuroblastoma cells were obtained from ATCC (CRL-2266) and maintained in a 1∶1 mixture of Eagle’s Minimum Essential Medium and F12 Medium with 10% serum. These cells were used to test neurotoxicity profiles of multiple HIV-1 isolates using HIV-1-infected PBMC supernatants. Approximately 250,000 cells were plated in 12-well plates and allowed to stabilize for 3–4 days. Three hundred microliters of HIV-infected PBMC supernatant with and without gp120 immuno-depletion were incubated with 600 µl of media containing the SH-SY5Y cells for a period of 24 hours. WST-1 assay (Roche) was used to determine neuronal viability in triplicate wells and percent cell death compared to untreated control cells was calculated.

#### BDNF measurement - gp120 treated Primary Human Neurons

Approximately 200,000 primary human neurons prepared as explained above were plated in 24-well plates and allowed to stabilize for 2 days and media replenished with no serum. The gp120 protein from HIV-1_Bal_, HIV-1_96ZM651_ and HIV-1_IN16055_, in PBS, were added to the neurons to obtain a final concentration of 0.5 nM (the reduced input of gp120 protein was in an attempt to minimize excessive cell death, which increases the level of soluble BDNF). Media was collected at 6 h and 24 h time points and BDNF ELISA (Promega) was performed on the supernatant according to manufacturer’s instructions. Results plotted are an average of 4 different ELISA measurements from two different sets of neuronal cultures.

## Results

### Intra-clade regional variation in HIV-1C envelope glycoprotein gp120

**Figure 1:**
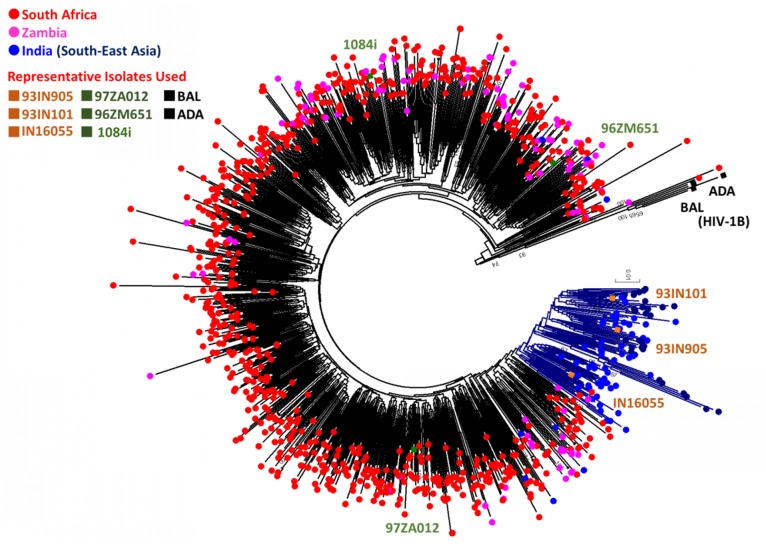
Phylogenetic analysis of gp120 sequences. Neighbor-joining phylogenetic tree was constructed in MEGA 6.06 software using 878 HIV-1C and four HIV-1B (as outlier) gp120 sequences from India and from the Southern African Countries of South Africa and Zambia. Due to the comparatively fewer HIV-1C gp120 sequences that were available from India, we included HIV-1C gp120 sequences from China and Myanmar that were available in the database. A separate phylogenetic tree with HIV-1C gp120 sequences from China, Myanmar confirmed their Indian origin. HIV-1B sequences from US were included in the phylogenetic analysis for comparison. Bootstrap values >50% were shown in the tree. The HIV-1C gp120 sequences from India and Southern African countries are genetically distinct and cluster separately. The specific isolates used in our in-vitro analysis (HIV-1_ADA_, HIV-1_Bal_, HIV-1_IndieC1_, HIV-1_93IN905_, HIV-1_IN16055_, HIV-1_1084i_, HIV-1_97ZA012_ and HIV-1_96ZM651_) are included in this analysis (and indicated on the diagram) to show that they qualify to be used as representative isolates.

Based on our previous findings that HIV-1C from Indian subcontinent has evolved independently and the fact that Indian clade C HIV-1 isolates have lower neurovirulence compared to HIV-1C from Southern African countries [18] we hypothesized that gp120 from South Asian countries should also be genetically divergent. To examine these differences, phylogenetic analysis was conducted using HIV-1 gp120 sequences from South Asian countries (mainly Indian isolates and sequences of Indian origin from China and Myanmar) and Southern African Countries (South Africa and Zambia) where HIV-1C is the predominant clade. Our analysis indicated that the two HIV-1C gp120s are genetically distinct and that the Indian HIV-1C gp120 sequences form a separate cluster segregating away from the Southern African HIV-1C sequences indicating they have evolved independently of each other (Figure 1). Previous molecular clock studies that measured the time of origin (tMRCA: time to most recent common ancestor) of the South African and Indian HIV-1C isolates using mutation rates and sequence diversity, have concluded that the South African and Indian HIV-1C have evolved independently of each other [25]. The intra and inter-HIV-1C sub-clustering pattern identified that the isolates from Southern Africa and India that were used in the in-vitro functional studies are well distributed (Figure 1) in the tree and qualify to be used as representative isolates.

#### Indian HIV-1C isolates have lower neurotoxicity compared to Southern African HIV-1C

**Figure 2:**
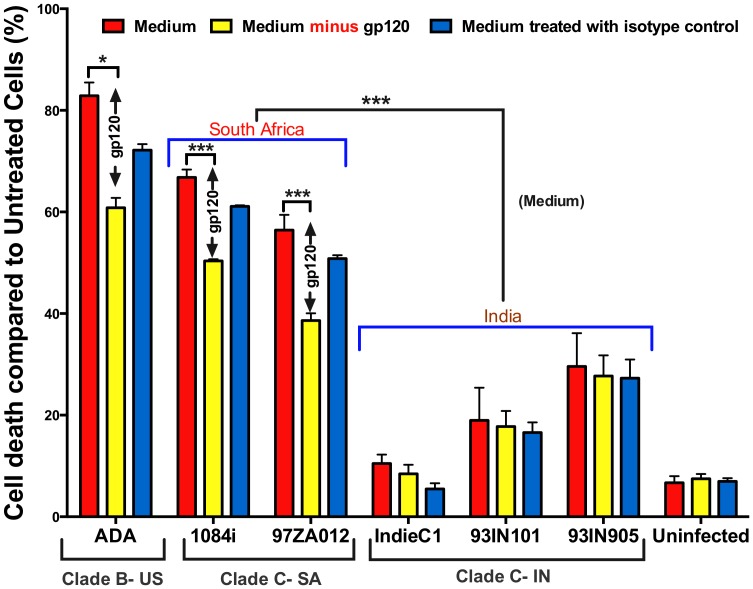
Differential neurotoxicity of Southern African and Indian HIV-1 clade-C isolates. **A**. gp120 of Indian HIV-1C isolates have minimal neurotoxicity. SH-SY5Y cells were incubated for 24 h with 50 µl of spent medium from PBMCs infected with representative isolates from (US HIV-1B, Southern African HIV-1C and Indian HIV-1C) with approximately similar viral loads. Supernatants were also immunodepleted of gp120 using anti-gp120 VRC03 (AIDS Repository) or Isotype Control IgG1 (BD) antibodies. WST-1 cell viability assay revealed US HIV-1B and Southern African HIV-1C supernatants caused significantly greater neuronal cell death compared to HIV-1C from India and a significant proportion of neuronal death is due to gp120. The absence of decrease in neurotoxicity upon neutralization of gp120 specifically in the Indian isolates was markedly different from the rest. Medium indicates untreated PBMC supernatants, while ‘Medium minus gp120’ refers to gp120 immunodepleted medium. An average of 2 Southern African isolates (medium) and of the 3 Indian isolates were compared. (*p<0.05; ***p<0.005).

We have previously shown that the Indian HIV-1C isolate HIV-1_IndieC1_ is less neurotoxic than the Southern African HIV-1_1084i_. Here, we tested the in-vitro neurotoxicity of multiple, representative Southern African (HIV-1_1084i_ and HIV-1_97ZA012_) and Indian HIV-1C isolates (HIV-1_IndieC1_, HIV-1_93IN905_ and HIV-1_93IN101_) along with a known neurovirulent US HIV-1B isolate (HIV-1_ADA_) to determine the contribution of gp120. We used HIV-1 infected PBMC supernatants with approximately equal viral loads and tested their neurotoxicity by measuring cell viability using SH-SY5Y neuronal cell line with and without immuno-depletion of gp120. Our results are striking in that all Indian isolates tested showed lower neurotoxicity than the US HIV-1B or Southern African HIV-1C isolates (Figure 2), which showed robust neurotoxicity. While gp120 immuno-depletion from spent medium of Indian isolates (Figure 2) caused only a slight diminution in neurotoxicity, immuno-depletion from that of Southern African isolates lead to a significant reduction in neurotoxicity. Even though the three Indian isolates tested were all less neurotoxic than the two Southern African isolates, we note that they still retain higher neurotoxicity than the uninfected PBMC media – suggesting the presence of neurotoxicity factors other than gp120.

#### Assessing the contributions of gp120 and Tat in HIV-supernatants to neurotoxicity

**Figure 3:**
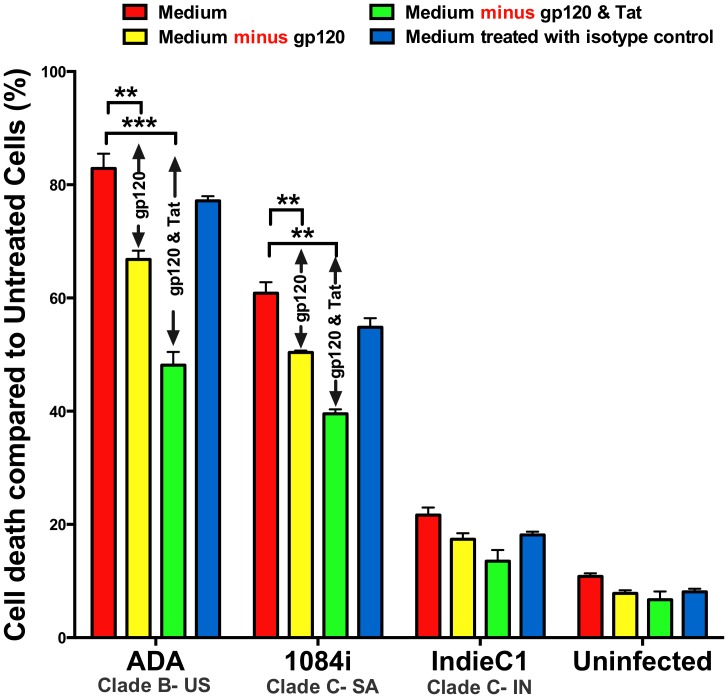
Relative contribution of gp120 and Tat to neurotoxicity. HIV-1-infected MDM supernatants (100 µl) from representative isolates US clade B (HIV-1B:HIV-1_ADA_), Southern African (HIV-1C:HIV-1_1084i_) and Indian clade C HIV-1 (HIV-1C:HIV-1_IndieC1_) with equal p24 loads were diluted in SH-SY5Y media and added to SH-SY5Y cultures and incubated for 24 h. Media with and without immuno-depletion of gp120 and Tat were tested. Medium indicates untreated MDM supernatants, while ‘Medium minus gp120’ and ‘Medium minus gp120 and Tat’ refer to gp120- immunodepleted and gp120 and Tat-depleted media respectively. WST-1 assay was performed to determine neuronal death. (**p<0.01; ***p<0.005).

Neuropathogenesis in HAND involves the exposure of neurons in the central nervous system specifically to HIV-infected macrophages. Therefore, we collected macrophage supernatants from HIV-1_ADA_ (US clade B), HIV-1_IndieC1_ (Indian clade C isolate) and HIV-1_1084i_ (Southern African clade C) with equal viral loads. We then immunodepleted gp120 alone or serially immuno-depleted both gp120 and Tat to evaluate the relative neurotoxic contributions of gp120 and Tat proteins. With respect to overall neurotoxicity of the untreated HIV-infected MDM supernatants, we observed a similar result as seen with PBMC supernatants: the neurotoxicity induced by MDM supernatant infected with HIV-1_IndieC1_ (≈ 21%) is significantly lower than that for either HIV-1_1084i_ (≈ 60%) or for HIV-1_ADA_ (≈ 82%) (Figure 3). However, upon combined immunodepeletion of Tat and gp120 from MDM supernatants, with both Clade B and Southern African clade C isolates, we see that the neurotoxicity is reduced by 35% to 40% - indicating that Tat and gp120 are responsible for a significant proportion of the neurotoxicity of the HIV-infected medium. The results also show that both Tat and gp120 of US clade B and Southern African clade C HIV-1 cause robust neurotoxicities. In contrast, in the case of Indian clade C HIV-1, the immuno-depletion of gp120 alone or of both gp120 and Tat, did not further reduce the already lower level of neurotoxicity. Combined with our earlier work, it appears that the overall reduced neurotoxicity of spent media from the Indian HIV isolates results from the reduced neurotoxicity of both Tat and gp120 proteins [18].

### Use of recombinant gp120 to confirm its role in regional variation in neurovirulence

**Figure 4:**
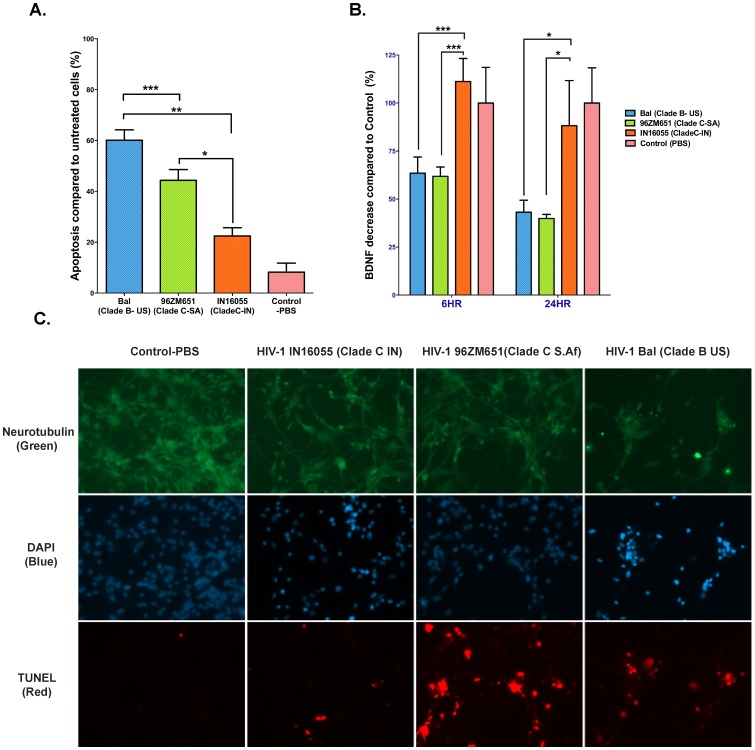
Recombinant gp120 proteins cause apoptosis in Primary Human neurons and reduce BDNF levels. **A.** *gp120 mediated neuronal apoptosis*. Primary Human Neurons were treated with 1 nM each recombinant gp120 s from representative US-B (HIV-1_Bal_), Southern African HIV-1C (HIV-1_96ZM651_) and Indian HIV-1C (HIV-1_IN16055_) isolates. Percent TUNEL positive cells from 20 different fields in 3x wells per treatment were analyzed and the level of apoptosis was determined. Similar to what was determined using immuno-depletion of gp120 in HIV-1-infected supernatants, the recombinant gp120 from Indian isolate showed a significantly lower percentage of neurotoxicity. **B.** *gp120-mediated effect on BDNF release*. Supernatants were collected from the primary human neurons following treatment with recombinant gp120s at 6 and 24 hours. Secreted BDNF levels were measured using ELISA and the results were plotted as a percentage of the Control (PBS). Both HIV-1_Bal_ (US clade-B) and HIV-1_96ZM651_ (Southern African clade-C) gp120s resulted in a 40% decrease in secreted BDNF levels at 6 h and a decrease of 60% at 24 h as opposed to HIV-1 IN_16055_ which showed no decrease at 6 h and a 10% decrease at 24 hours compared to Control (PBS). **C.** *Neuronal apoptosis caused by recombinant gp120 proteins.* Tunnel stained primary human neurons co-stained with anti-neurotubulin antibodies and DAPI (Prolong Gold anti-fade with DAPI, Invitrogen) to verify the direct effect of recombinant gp120 on neuronal death. The four panels show representative fields for neurons treated with PBS and 1 nM recombinant gp120 s from Indian HIV-1C, Southern African HIV-1C and US-B, isolates. Neurons treated with Southern African clade C gp120 and US- Clade B show greater neurotoxicity. (*p<0.05; **p<0.01; ***p<0.005).

In order to confirm the contribution of gp120 towards neurotoxicity differences observed with the MDM-media infected by HIV-1 isolates from different regions, we obtained recombinant gp120 proteins from representative isolates. We treated primary human neurons with 1 nM each of gp120 derived from HIV-1_Bal_ (US clade-B), HIV-1_96ZM651_ (Southern African clade-C) and HIV-1_IN16055_ (Indian clade-C) isolates for a period of 24 hours and then calculated the percent apoptosis based on the proportion of DAPI stained primary human neurons that were TUNEL-positive. We could replicate the same neurotoxicity profile that we observed using spent medium, in that the US clade-B HIV-1 (HIV-1_Bal_) and the Southern African clade-C HIV-1 (HIV-1_96ZM651_) gp120 resulted in 60% and 40% cell death respectively compared to the Indian clade-C HIV-1 (HIV-1 _IN-16055_) gp120, which showed less than 20% neurotoxicity (Figure 4A and 4C). It is important to note that the isolates from which the recombinant gp120s were derived are distinct from the isolates used to test the neurotoxicity of MDM-infected medium. Therefore, these results further validate our finding that intra-clade regional variations seen in neurotoxicity of HIV-1 clade-C isolates are attributable to the nature of their respective gp120s.

### BDNF levels in recombinant gp120 treated primary human neurons

It has been shown that gp120 drives neurotoxicity by inhibiting the processing of pro-BDNF (brain derived neurotrophic factor) to BDNF or by decreasing the expression of BDNF. This inhibition can be measured by a reduction in the levels of secreted BDNF [26]. Based on our results above, we surmised that the differences in neurotoxicity of the different gp120 s will be reflected in BDNF secretion levels. Therefore, we measured BDNF levels in the neuronal culture media after incubation with gp120 only as another indicator of neurotoxicity. We measured the effect of recombinant gp120 s derived from HIV-1_Bal_ (US clade-B), HIV-1_96ZM651_ (Southern African clade-C) and HIV-1_IN16055_ (Indian clade-C) isolates on BDNF levels at 6 hours and 24 hours post-incubation with primary human neurons. Both HIV-1_Bal_ (US clade-B) and HIV-1_96ZM651_ (Southern African clade-C) gp120s resulted in approximately a 40% decrease in secreted BDNF levels at 6 hours and a decrease of 60% at 24 hours as opposed to Control (PBS) (Figure 4B). At the same incubation intervals, gp120 derived from the Indian clade-C HIV-1_IN16055_ showed minimal effect on secreted BDNF levels suggesting it does not interfere with BDNF processing. It has been shown that in patients suffering from severe form of HAND (HAD), BDNF levels are significantly reduced in the brain [26]. Thus, our *in vitro* observations of a lack of effect on BDNF levels upon treatment with gp120 of Indian isolates support our conclusion that gp120 is one of the key determinants of lower neurovirulence underlying the decreased prevalence of HAD in this geographical region.

## Discussion

The envelope glycoprotein gp120 of HIV-1 is a major neurotoxicity determinant. In this report, we have shown that the robust neurotoxicity of HIV-1 gp120 is not universally observed in all clade C HIV-1 isolates. The isolates from Southern Africa revealed robust neurotoxicity similar to US clade B HIV-1 gp120 proteins, while gp120 from HIV-1C isolates from India showed minimal neurotoxicity. We have employed HIV-1C-infected supernatants derived from different isolates to demonstrate differential neurotoxicity and verified the differential effect by immunodepleting gp120 to demonstrate a specific role for gp120. We further corroborated this finding using purified gp120 from various strains, which shows that even when equal amounts of gp120 of different strains is employed, differential neurotoxicity is observed (Figure 4A). HIV gp120 from clade B HIV-1 directly binds NMDAR on neurons and can cause a lethal influx of calcium ions [19] and membrane depolarization [27]. Clade-B gp120 drives the production of excess glutamate from neurons [28], and also causes NMDAR clustering on the cell surface and prevents receptor internalization, exacerbating the problems from NMDAR over-activation [29]. Our findings will now allow us to further examine mechanistic differences in these pathways between the two sets of intra-clade C isolates in their ability to cause neurotoxicity.

Previous studies that have attempted to identify viral determinants of differential neurovirulence of the Indian HIV-1C and the US HIV-1B isolates have focused on Tat protein. The premise of those studies has been that the decreased monocyte migration caused by C31S polymorphism in Tat prevents infiltration of HIV-infected monocytic phagocytes into brain which forestalls additional events that cause inflammatory insult due to viral proteins and host cytokines in the CNS. In addition, the dicysteine motif of Tat is critical for interaction with NMDAR receptor on the neurons [13]. Therefore, until now, the decreased neurovirulence of HIV-1C has been primarily attributed to C31S polymorphism. In this study, we have shown that in addition to Tat protein, the Indian HIV-1C owes its reduced neurovirulence also to the low neurotoxicity of its gp120. We also show that the gp120 proteins encoded by Southern African HIV-1C isolates have robust neurotoxicity in contrast to Indian HIV-1C isolates. Thus, considering that about 20–25% of Southern African HIV-1C isolates have an intact dicysteine motif in Tat, one could envision that the presence of Tat dicysteine motif in a given isolate will trigger monocyte infiltration of HIV to brain that can increase viral load in the brain as well as bring additional inflammatory insults due to the presence of more neurotoxic gp120. In the case of Southern African isolates with a C31S substitution in Tat, in spite of the presence of a neurotoxic gp120, the likelihood that it would appear in the brain would be significantly reduced. In contrast, in the case of Indian HIV-1C isolates, not only does the absence of a dicysteine motif due to nearly universal C31S polymorphism in India reduce the potential for brain infiltration, but the absence of neurotoxicity due to gp120 may also contribute to reduced neurovirulence. Thus, our results provide new insights into why HAD incidence in Southern African countries such as South Africa or Zambia is much higher than that in India.

The Indian HIV-1C is known to have originated from South Africa [25]. Previous phylogenetic studies have concluded that the Indian HIV-1C has been evolving independently of the South African HIV-1C due to selection forces involving host genetics and host immune responses that are distinct in the Indian population [30]. Shankarappa *et al*. examined 192 gp120 sequences from 21 individuals in India and found that nearly all sequences form a phylogenetically distinct lineage within subtype C, which they designated C(IN). Similarly, our previous phylogenetic analysis of full-length molecular clones of HIV-1C as well as Tat sequences from India, Botswana, South Africa and Zambia [18] showed that the Indian HIV-1C clusters away from Southern African HIV-1C. In this report, we have compared the gp120 sequences of isolates from Southern African countries (Zambia and South Africa) with the Indian HIV-1C gp120 sequences, and we have shown that sequences from these two regions have significant genetic differences and cluster into separate groups. Our finding that gp120 of Indian HIV-1C lacks neurotoxicity, while that in Southern Africa exhibits robust neurotoxicity is in agreement with the results of phylogenetic analysis. Although the Southern African and Indian HIV-1C may have a common origin, their independent evolution under different sets of host selection processes may have resulted in the emergence of distinctive sets of properties in HIV-1C from these two regions. It is likely that during this independent evolution, the Indian HIV-1 gp120 has evolved into a non-neurovirulent form. It is also possible, although less likely, that the clade C HIV-1 started as a non-neurotoxic clade and the Southern African Clade C evolved into a more neurotoxic clade subsequent to its spread to India. These notions can be tested only after a specific motif responsible for neurotoxicity is identified in the Southern African clade C HIV-1 gp120 to facilitate studies to examine its origin or loss during the evolution of clade C HIV-1. The findings reported here thus provide impetus to further stimulate research in this important area of neuroAIDS research.

These findings, combined with the fact that HIV-1C Tat protein is also distinctly different from that of Southern African HIV-1C in terms of its neurovirulence properties suggest that the Indian HIV-1C may have evolved to be less neurovirulent in general. It is unclear, with our present knowledge, whether neurovirulence is an unintended consequence of HIV-1 evolving under the pressure to be a successful pathogen infecting the peripheral immune system. However, our finding that intra-clade variation in the evolution of HIV-1C leads to differential neuropathogenesis provides us with new opportunities to examine this question.

## Conclusions

We previously showed the presence of specific neurovirulence determinants in Tat protein of Southern African HIV-1C isolates and hypothesized that it is possible to predict the neurocognitive outcomes of infection by HIV-1C by genotyping HIV-1 for specific Tat signatures early in HIV-1 infection. In the current work, we have shown preliminary evidence that specific neurovirulence signatures also exist in gp120 of clade C that is not universally present in clade C HIV-1 in all regions. Additional work can help us identify these signatures, which may allow one to predict the neurocognitive outcome in millions of HIV-1C infected individuals by genotyping both Tat and gp120 of HIV-1C isolates in those patients.
